# Population genomics identifies Italian and North American origins of *Popillia japonica* in Switzerland

**DOI:** 10.1038/s41598-026-39440-4

**Published:** 2026-02-12

**Authors:** Chiara Pedrazzini, Rebecca Funari, Claudio Cucini, Francesco Nardi, Giselher Grabenweger, Franco Widmer, Jürg Enkerli

**Affiliations:** 1 Molecular Ecology, Agroscope, Zürich, Switzerland; 2 Dipartimento Di Scienze Della Vita, Università Di Siena, Siena, Italy; 3 NBFC, National Biodiversity Future Center, Palermo, Italy; 4 Extension Arable Crops, Agroscope, Zürich, Switzerland

**Keywords:** Japanese beetle, Population genomics, Genetic structure, Biological invasion, Ecology, Ecology, Evolution, Genetics

## Abstract

The Japanese beetle (*Popillia japonica*), native to Japan, is a major invasive species in North America and Europe. Its colonization of Europe began on the Azores (1970s), followed by Italy (2014) and southern Switzerland (2017). In 2023, the presence of the pest was reported in three areas of Switzerland: Kloten (Canton of Zürich, north-eastern Switzerland), Basel (Canton of Basel-Landschaft, northern Switzerland), and in eastern Canton of Valais (South Switzerland) near the Italian border. In 2024, *P. japonica* individuals were discovered in several other Swiss Cantons. To trace *P. japonica* spread and origins in Switzerland, we investigated 42 individuals from infested areas, comprising scattered findings across Switzerland. Phylogenetic and population structure analyses using whole-genome resequencing, including data of previously sequenced samples from Japan, North America, the Azores, Italy, and Southern Switzerland, revealed distinct *P. japonica* migration patterns. Populations in Basel, Valais, and central Switzerland likely originated from Ticino/Northern Italy via road or rail transport. Conversely, the population near Zürich Airport was identified as an independent introduction from North America, likely through unintentional air transport. These findings offer insights into *P. japonica* spread across Switzerland, highlighting the need for enhanced monitoring and identification of invasion pathways.

**Supplementary Information:**

The online version contains supplementary material available at 10.1038/s41598-026-39440-4.

## Introduction

*Popillia japonica* Newman, 1841 (Coleoptera: Scarabaeidae: Rutelinae), commonly known as the Japanese beetle, is native to Japan, where it occurs across the islands of Honshu, Hokkaido, Kyushu, and Shikoku^1^. Over the past century, *P. japonica* has become a relevant invasive pest in both North America and Europe^2,3^. With the ongoing effects of climate change and rising temperatures, the species is expected to further expand its range into northern regions of both continents, posing an increasing threat to agriculture and ecosystems^2^.

The Japanese beetle is a polyphagous insect that feeds on over 400 plant species, including key crops like grapes, maize, soy, and fruit trees, with adult beetles causing extensive damage to foliage, flowers, and fruits of host plants^4^. In Europe, unmanaged infestations of *P. japonica* are projected to result in annual damage costs ranging from €30 million to as much as €7.8 billion, particularly affecting crops such as grapes,  maize, soy, apples, peaches, and cherries^5^. Due to its rapid spread and the plant damages caused by the adults, the Japanese beetle has been included on the EU Priority Pest List^6^ and designated as a quarantine organism in Switzerland by the Federal Office for Agriculture [FOAG;^7^.

**Fig. 1:**
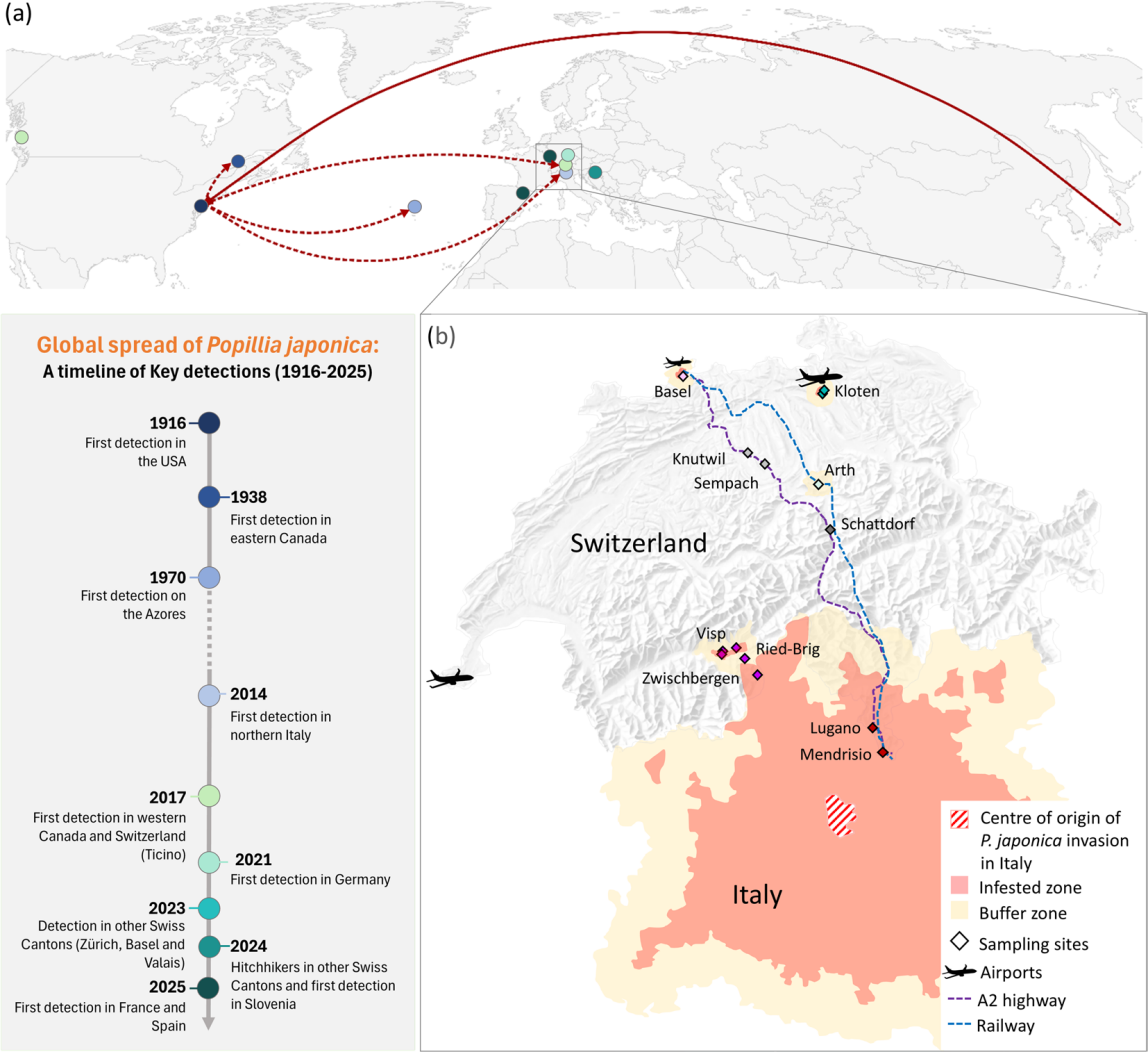
Global and regional spread of *Popillia japonica*. (A) Global invasion pathway of *P. japonica*, showing primary introductions from Japan to North America (1916), secondary spread to eastern Canada (1938), the Azores (1970s) and Italy (2014), and subsequent establishment in southern Switzerland (2017) and further expansion across Europe (2023–2025). The timeline summarizes key invasion stages. (B) Map depicting infested zones of *Popillia japonica* in Switzerland and Italy (as of April 2025), along with the beetle collection sites across Switzerland used in this study. Site labels correspond to those listed in Table 1. The centre of origin of *P. japonica* invasion in Italy as well as areas defined as buffer zones with increased surveillance, but no reported presence are also indicated. Swiss collection sites span the following Cantons and municipalities: Ticino (Lugano and Mendrisio), Zürich (Kloten), Basel-Landschaft (Basel), Valais (Visp, Ried-Brig, and Zwischbergen), Luzern (Knutwil and Sempach), Schwyz (Arth), and Uri (Schattdorf). Major Swiss airports are marked with airplane symbols, highlighting key international hubs such as Zürich (Kloten) and Geneva, as well as the smaller Basel airport. The A2 highway and the railway line—major north–south corridors connecting the Canton of Basel with the Canton of Ticino—are also depicted. Maps were generated using the R packages ggplot2 3.5.1 (https://ggplot2.tidyverse.org), sf 1.0–15 (https://r-spatial.github.io/sf/), rnaturalearth 1.0.1 (https://docs.ropensci.org/rnaturalearth/), and raster 3.6–26 (https://rspatial.org/raster).

Historical and phylogenetic records have traced the invasive dispersal pathways of *P. japonica* from its native range. The species was first detected in North America near Philadelphia in 1916, likely introduced via soil of imported Japanese *Iris* spp. rhizomes, and rapidly spread across the eastern United States (USA) and Canada despite extensive control efforts (Fig. 1a,^1,8–10^. Recent studies ^11,12^ have demonstrated that populations of *P. japonica* in the United States, originated from Northern or Central Japan, and that the invasion in Canada subsequently resulted from secondary spread of the USA populations (Fig. 1a ^11,12^.

In Europe, *P. japonica* was first detected in the 1970s on Terceira Island, Azores, near the Lajes North America Air Base (Fig. 1a;^13^. Previous phylogeographic studies have suggested it was introduced from the USA, likely via unintentional air transport ^11,12,14^. Despite substantial eradication efforts, the beetle became established^13^. Currently, *P. japonica* is established on all Azorean islands, except Santa Maria^15^.

In July 2014, the first observation of *P. japonica* in Italy has been reported (Fig. 1a), just a few kilometers from Milano International Malpensa Airport and Cameri Military Airport^16^. Shortly afterwards, an outbreak was confirmed in the Ticino Natural Regional Park in Northern Italy, and since then, the beetle has spread in all directions around the site of its initial observation into the Piemont and Lombardy regions^17^. Prior genetic investigations have revealed that the introduction to Italy originated from the USA (Fig. 1a), most likely, once again, via airplane transport^11,12,14^. In order to contain the infestation, comprehensive control strategies were employed^18,19^, along with risk-based protocols at the Malpensa and Cameri airports^20^. However, despite these considerable efforts, the insect has widely spread and has reached southern Switzerland (Canton of Ticino, Fig. 1) in 2017, near the Italian border [Stabio;^21^]. Genetic analyses have confirmed that the *P. japonica* population established in the Swiss Canton of Ticino represents an expansion of the Northern Italian population^11,12,14^. Since its first establishment in 2017, *P. japonica* each year has spread further north in the Ticino region^22^.

In the following years, *P. japonica* has been detected in additional sites in Switzerland, including new outbreaks in the Cantons of Zürich, Basel, Valais, as well as individual catches in other Cantons, such as in the Cantons of Aarau, Grisons, Luzern, Schaffhausen, Schwyz, Solothurn and Uri (Fig. 1b). In particular, in July 2023, a new population of *P. japonica* was detected in Kloten (Canton of Zürich) near Zürich Airport, representing the first record of a population north of the Alps^23^. In 2023, two *P. japonica* individuals were also collected from a trap at the city limit of Basel approximately 100 km north-west of Zürich Airport, but these were considered hitchhikers rather than evidence of an established population, as no other beetles were found after further searches (Giselher Grabenweger, Agroscope, personal communication). In the same summer, *P. japonica* presence was confirmed in the Simplon area in Canton of Valais, close to the Italian border (Fig. 1b;^24^.

In order to eradicate the still small and restricted population in Kloten, several control strategies were implemented. These included direct chemical treatments and pheromone traps combined with insecticides (e.g., deltamethrin) to target adults, irrigation bans to prevent egg deposition, mechanical soil disturbance, and the precautionary application of nematodes to control larval populations^23^. Concurrently, monitoring and preventive measures in Basel were intensified, involving increased trap density, shortened inspection intervals, and the application of nematodes to control larvae populations^25^. Similarly, in Valais, trap density was increased, and inspection intervals were shortened (Joana Weibel, Agroscope, personal communication).

Despite control and preventive measures in Kloten and Basel, relevant numbers of *P. japonica* individuals were captured at both sites in 2024, confirming the presence of two distinct populations^26^. In addition, in summer 2024 *P. japonica* was detected in other parts of Switzerland, including an additional infestation area in the Canton of Valais^24^, as well as isolated captures in various other Cantons, that is in the Cantons of Aarau, Grisons, Luzern, Schaffhausen, Schwyz, Solothurn and Uri (Joana Weibel, Agroscope, personal communication).

Climatic simulations predicted that regions currently affected by *P. japonica* in Switzerland provide optimal conditions for the pest survival, with rising temperatures expected to render the Swiss Central Plateau increasingly suitable by the end of the century^2^. These findings highlight specific risks for Switzerland and align with broader European trends, where interceptions of *P. japonica* are steadily increasing, as exemplified by recent reports from Germany^27^, Slovenia^28^, France and Spain^3^ that underscore its rapid geographic expansion.

Recent statistical models, allowing to evaluate the risk of *P. japonica* introduction from ongoing outbreaks in Northern Italy and Ticino, have indicated that the area of Zürich might be particularly vulnerable to invasions of *P. japonica*, due to its extensive transportation networks, including air, rail, and road connections^29^. Consequently, the potential sources of the invasion in the Kloten area may include migration from outbreak regions in Ticino and Italy, as well as possible introductions through Zürich International Airport from infested areas in the Azores, North America, or Japan.

This study aims to trace the spread and origins of *P. japonica* in Switzerland with population genetic structure analyses, with a focus on regions including Kloten and Basel, and incorporating individuals collected from various Swiss cantons such as Ticino, Valais, Uri, Luzern and Schwyz. This approach enables an examination of the species progressive colonization and the influence of human-mediated introductions—such as those near Zürich International Airport—that may initiate new outbreaks. By differentiating between gradual range expansions and isolated introductions potentially leading to new infestations, the research provides deeper insights into the dispersal pathways of this invasive species.

## Results

After filtering, 3,383,671 SNPs (22.7% of unfiltered sites) were retained. Examination of individual-level metrics did not reveal any outliers, and all individuals were retained for subsequent analyses, resulting in a full dataset of 125 *P. japonica* individuals, including 42 *P. japonica* individuals from this study (Table 1) alongside 83 samples from Funari et al.^11^. Linkage disequilibrium pruning resulted in a final dataset of 317,994 unlinked SNPs (9.4% of filtered sites).

**Table 1:** Sampling sites, year, number of individuals (N) and coordinates of novel *Popillia japonica* collections. Canton corresponds to the Swiss federal state, and Municipality corresponds to local administrative units within each canton. A map of sampling site is represented in Fig. 1. The coordinate system used was WGS84.

Number of the collection	Canton	Municipality	Year of collection	N *P. japonica* individuals	Coordinate latitude	Coordinates longitude
1	Zürich	Kloten	2023	1	47.456826	8.57951
2	Zürich	Kloten	2023	7	47.456516	8.58446
3	Zürich	Kloten	2024	8	47.456516	8.58446
4	Basel-Landschaft	Basel	2023	1	47.533752	7.620478
5	Basel-Landschaft	Basel	2024	9	47.533752	7.620478
6	Valais	Visp	2024	1	46.298506	7.874226
7	Valais	Visp	2024	1	46.291836	7.87658
8	Valais	Ried-Brig	2024	1	46.318707	8.013278
9	Valais	Ried-Brig	2024	1	46.280692	8.036575
10	Valais	Zwischbergen	2024	4	46.191214	8.142858
11	Ticino	Lugano	2023	3	45.950137	8.909968
12	Ticino	Mendrisio	2023	1	45.847586	8.957109
13	Uri	Schattdorf	2024	1	46.848409	8.633831
14	Luzern	Sempach	2024	1	47.145164	8.184519
15	Luzern	Knutwil	2024	1	47.197437	8.066559
16	Schwyz	Arth	2024	1	47.048766	8.554517

### Analyses of population structure

**Fig. 2:**
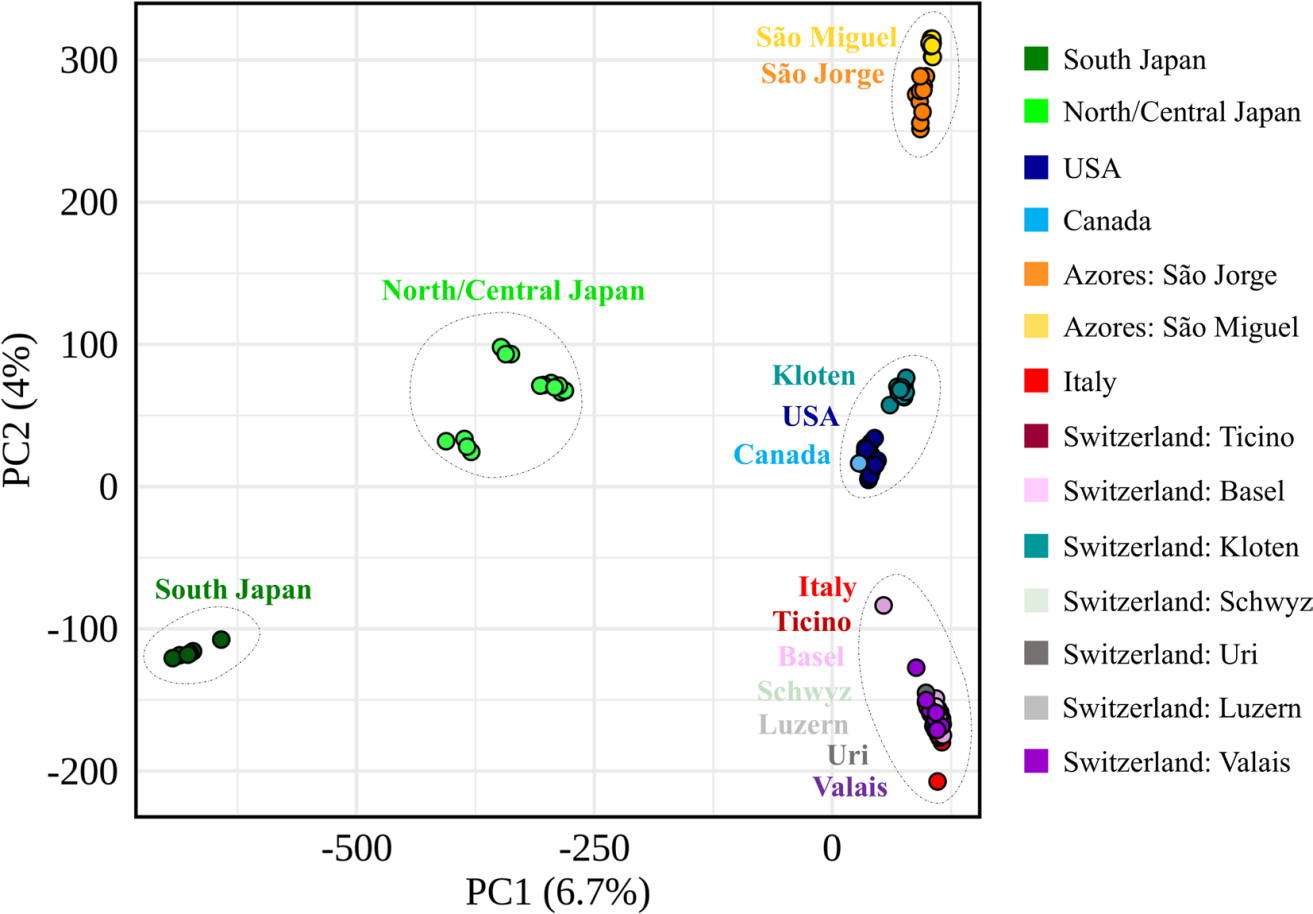
Principal component analysis (PCA) based on 317,994 SNPs from 125 *Popillia japonica* samples collected across different regions worldwide: South and North/Central Japan, USA, Canada, the Azores (São Jorge and São Miguel), Italy, and Switzerland (Ticino, Basel, Kloten, Schwyz, Uri, Luzern, and Valais). Regions are labelled and enclosed within ovals representing the clusters identified through PCA analysis.

The principal component analysis (PCA) identified five distinct, main genetic clusters (Fig. 2). These clusters included: (1) South Japan, (2) North/Central Japan, (3) the two Azorean Islands (São Jorge and São Miguel), with some genomic differentiation between the islands, (4) a cluster comprising samples from the USA, Canada, and Kloten, and (5) a cluster representing samples from Italy and from various regions in Switzerland (Ticino, Basel, Valais, Luzern, Schwyz, and Uri; Fig. 2). The first principal component (PC1), explaining 6.7% of the variance, separated South and North/Central Japan from the invasive populations, while the second component (PC2), accounting for an additional 4%, further divided the invasive populations into three groups: the Azores, USA/Canada/Kloten, and Italy/Switzerland (Fig. 2).

**Fig. 3:**
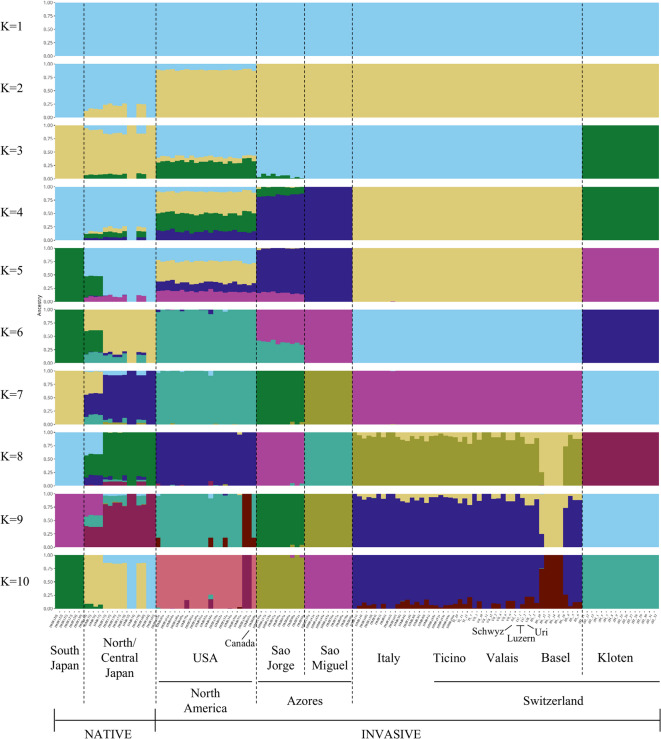
Admixture plots of 125 *Popillia japonica* individuals based on 317,994 SNPs and obtained from ADMIXTURE from K = 1 to K = 10. Each vertical line represents an individual, and the colour is proportional to the membership coefficient (Y-axis) to the K clusters. Geographic sites of origin are indicated at the bottom of the figure.

Genetic cluster analyses based on the software ADMIXTURE identified K = 5 as the optimal number of clusters, i.e., ancestral populations, based on the cross-entropy criterion showing the lowest value (Supplementary Figure S1). However, varying values of K revealed distinct population structures (Fig. 3). For instance, at K = 2, all samples from invasive regions were assigned to the same genetic cluster, while samples from Japan exhibited similar ancestry coefficients to a second cluster (Fig. 3). At K = 5, corresponding to the optimal number of ancestral populations, further differentiation became evident. Notably, South Japan separated from North/Central Japan, the Azores formed a distinct cluster showing admixture with North American populations, and Kloten samples exhibited co-ancestry with populations from North American (USA and Canada; Fig. 3). At K = 8, finer genetic structure emerged: the two Azorean islands (São Jorge and São Miguel) formed distinct clusters, some Basel samples formed a new cluster, and two USA samples were assigned to a distinct group, indicating further divergence within North America and Europe, while samples from Italy and Swiss regions showed similar ancestry proportions (Fig. 3).

While positive overall, the Mantel test did not identify a significant correlation between genetic and geographic distances when performed specifically on populations from the invasive range, thus not providing evidence of isolation by distance (IBD; Supplementary Figure S2).

Kinship analysis revealed a high level of genetic relatedness in recently established, invasive populations (i.e. Italy and Ticino, Basel and Kloten; Supplementary Figure S3). Furthermore, within-population kinship was notably higher in invasive populations compared to their respective source populations, as expected following recent invasion. Focusing on kinship between populations, Basel displayed a closer relationship with Italy and Ticino than with the USA (Figure Supplementary S3). The Kloten population, in turn, showed closer relationship with USA, than with both Italy and Ticino, and Basel, although with marginal differences (Supplementary Figure S3).

### Phylogenetic analyses

**Fig. 4:**
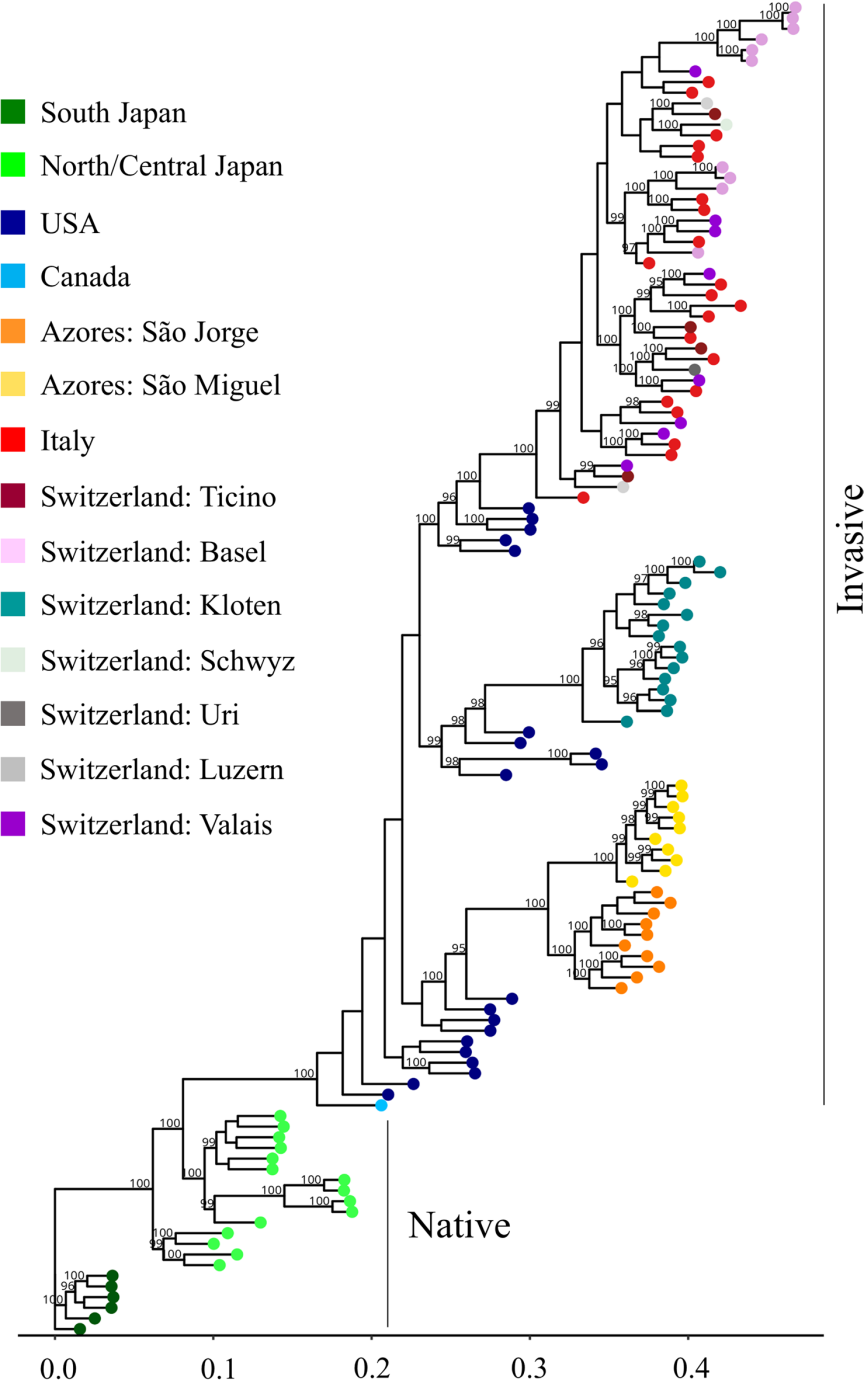
Phylogenetic tree of 125 *Popillia japonica* individuals constructed using the maximum likelihood algorithm. The relationships between clades are shown with reference to South Japan. The analysis includes samples from South Japan, North/Central Japan, the USA, Canada, the Azores, Italy, and Switzerland (Ticino, Basel, Kloten, Schwyz, Uri, Luzern, and Valais). Node dots are color-coded by geographic site of origin, and bootstrap values > 95% from 2,000 replicates are displayed at major nodes. The scale bar represents branch length in terms of the number of substitutions per site.

ModelFinder identified TVM + F + R6 as the best-fit nucleotide substitution model according to the Bayesian Information Criterion (BIC), and this model was subsequently used for phylogenetic reconstruction. Phylogenetic analyses including the 125 *P. japonica* individuals collected across various native and invasive areas identified several distinct clades. Samples from South Japan were positioned at the base of the phylogenetic tree (Fig. 4). All samples from invasive populations formed a single monophyletic clade derived from North/Central Japan, confirming earlier studies^11,12^. Within this clade of invasive areas, the USA samples formed a large paraphyletic group relative to three distinct invasive lineages: the Azorean samples (divided into São Jorge and São Miguel subclades), the Kloten samples, and a larger clade that included all the samples from Italy and Switzerland, with the exception of those from Kloten. This group comprised individuals from Ticino, Valais, Luzern, Schwyz, Uri, as well as Basel. While no clear phylogenetic clustering patterns were observed within this clade based on geographic origin, six of the ten samples from Basel formed a small distinct subgroup (Fig. 4).

### Demographic inference

**Fig. 5:**
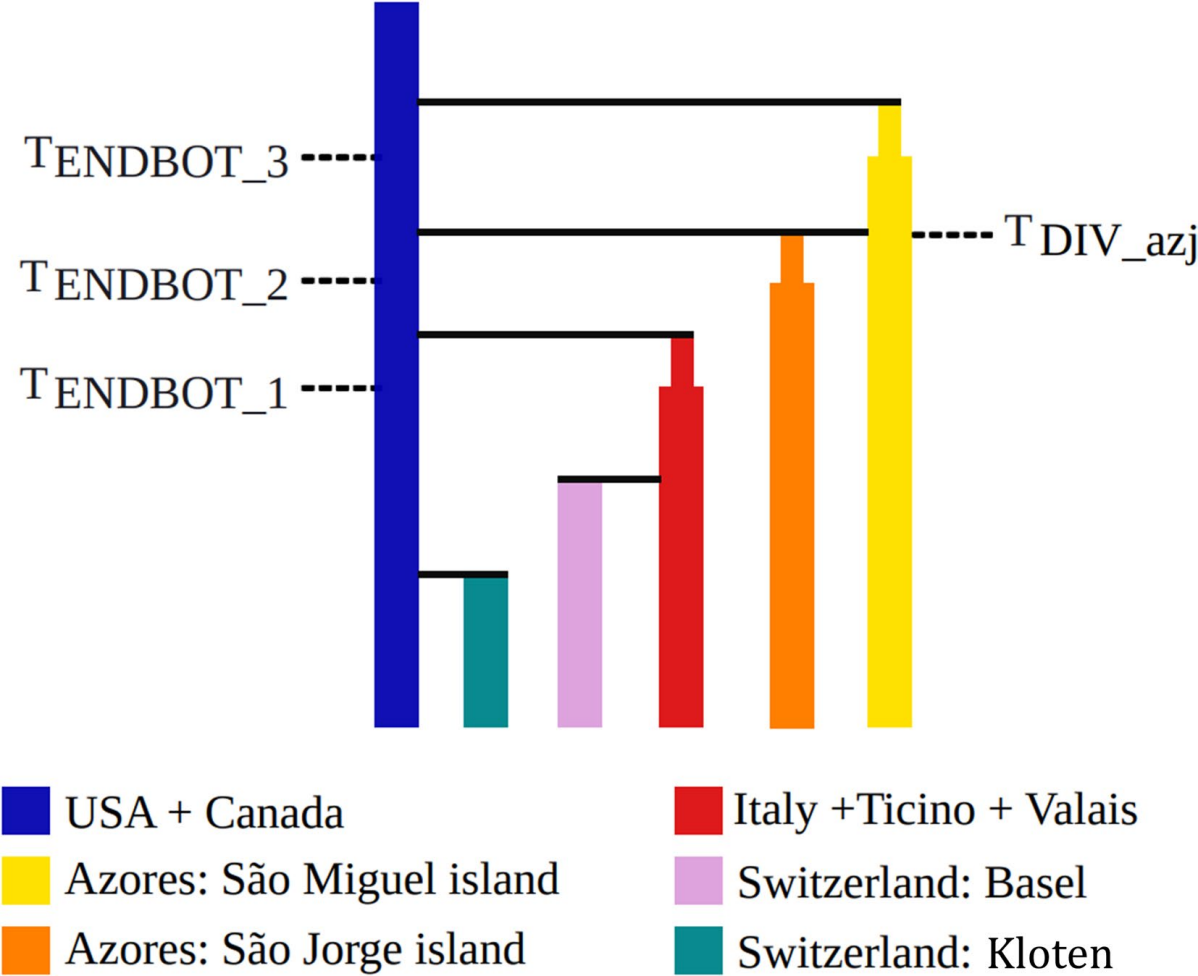
Best-supported demographic model inferred from demographic inference analyses. T_DIV_, indicates the divergence time of the admixed lineages. _Azj_, refers to the São Jorge population (Azores). T_ENDBOT_, denotes the end of a bottleneck event.

The demographic analyses, used to infer population-level historical and evolutionary processes, identified Model 1 (AIC: 4511.486) as the best-supported scenario for explaining the origin of *P. japonica* populations in Basel and Kloten (Fig. 5, Supplementary Table S2, Supplementary Figure S4, Supplementary Figure S5). This model posits that the Basel population has originated from the infestation in Italy, Ticino and Valais (collectively considered as a single source population), while the population in Kloten has derived from the infestations in the USA + Canada, entailing an independent introduction for the two Swiss populations. While clearly inferior, the AIC values of models 30, 24, 26, and 31 (4515.877, 4516.175, 4516.263, and 4516.692, respectively) remain relatively close to that of the best-supported model, falling within a range of marginal credibility (Supplementary Table S2, Supplementary Figure S5). Notably, all these models represent slight variations of Model 1, incorporating admixed populations such as Italy + Ticino + Valais, the USA + Canada, and/or Basel (specifically for Kloten) as potential source populations (Supplementary Figure S4). Such signals of admixture are consistent with expectations for very recent invasion events, as observed in the case of *P. japonica* (Supplementary Table S2, Supplementary Figure S4, Supplementary Figure S5).

## Discussion

This study provides an extensive investigation into the geographic origin of the Japanese beetle in Switzerland, with a focus on the two most severe infestations, located in Kloten (Canton of Zürich, northeastern Switzerland), and Basel (Canton of Basel-Landschaft, northern Switzerland). Additional individuals were included from infestations in the Southwest of Switzerland (Visp, Ried-Brig and Zwischenbergen, Canton of Valais), and in central Switzerland, where only a few single detections have been reported (Sempach and Knutwil, Canton of Luzern; Arth, Canton of Schwyz, and Schattdorf, Canton of Uri). Our research identified two major introduction pathways: (1) a direct introduction from North America into Kloten, likely via air transportation, and (2) a regional spread from Northern Italy and Ticino northwards into Valais, Luzern, Schwyz, Uri, and Basel, likely facilitated by human-mediated road or rail transport.

The here performed population structure and demographic analyses provided strong evidence that the *P. japonica* population in Kloten originated from an independent North American introduction. The distinct genetic separation of this population from all the other populations in North Italy and Switzerland, the demographic inference modeling, together with the fact that the population was detected initially near Zürich Airport in July 2023^23^, strongly suggest that the North America population may have acted as a central hub for secondary invasions into Europe, consistent with the *bridgehead effect*^30^, whereby an established invasive population facilitates further colonization events.

Borner et al.^29^ have assessed the potential spread of *P. japonica* across Europe and identified Zürich as a crucial hub accessible from the infested region spanning Northern Italy and Southern Switzerland through various modes of transportation (i.e., road, air and rail). Further, Zürich is a major international hub with direct flights from North America and Japan, highlighting its susceptibility to transcontinental introductions—a hypothesis formed by Borner et al.^29^, which is in agreement with the patterns observed in this study.

The detection and occurrence of *P. japonica* close to the Zürich Airport in Kloten may exemplifies the critical role of global air transportation networks in facilitating the spread of invasive species, as has been described by previous studies^31,32^. Nardi et al.^12^ and Strangi et al.^14^ have attributed previous introductions of *P. japonica* in the Azores and Italy to air traffic from North America, underscoring the role of international air travel in pest insect dispersal. Despite stringent biosecurity measures in North America, especially in the USA, including visual inspections, flight schedule adjustments, and plant transport regulations, the introduction to Kloten reveals persistent limits in preventing air transport-mediated invasions^33^.

Interestingly, while our results unequivocally support the direct introduction of the Kloten population from North America, there is evidence of minor genetic differentiation between this population and its source. This subtle divergence, observed in admixture and PCA analyses, underscores the complex dynamics of invasive populations, likely driven by extremely severe bottleneck events during the invasion process.

In addition to the best-supported scenario, in which the Kloten population originates from North America, demographic analyses identified four alternative scenarios with ΔAIC values within a marginal credibility range from the top model. These scenarios involve admixture for both Kloten and Basel, with potential source populations including Italy, Ticino, Valais, the USA, Canada, and Basel itself (specifically for Kloten; Supplementary Table S2, Supplementary Figure S4, Supplementary Figure S5). While these admixed scenarios cannot be completely excluded, they are not supported by the results of population structure analyses (i.e., PCA and ADMIXTURE), the kinship analyses, and the phylogenetic reconstruction. In combination with the restricted geographic range, the small sample sizes of the populations, and their relatively short period of coexistence, such admixed scenarios remain unlikely. An alternative explanation for the observed pattern is recent divergence of both the Kloten and Italian populations, with the genetic similarity reflecting shared ancestry rather than true admixture. In this case, both populations may have originated from a common source, with insufficient time since divergence to establish distinct genetic signatures. However, given the high level of co-ancestry and the brief time since separation, these patterns could easily be misinterpreted as evidence of intermixing. Overall, the scenario not including admixture remains the more plausible explanation.

The populations in Basel, Valais, and the single captures in Luzern, Schwyz, and Uri are likely the result of an ongoing spread originating from Ticino and Northern Italy. Notably, Basel has been identified by Borner et al.^29^ as one of the most accessible cities from these infested regions, exemplifying the pivotal role of regional road and train infrastructure in facilitating the beetle spread. Even more strikingly, the alignment of single captures in Luzern, Schwyz, and Uri with major transport routes, including the A2 motorway—Switzerland’s primary north–south axis connecting Basel to Chiasso (Canton of Ticino)—and the Lugano-Basel railway (Fig. 1b), may emphasize the profound impact of human-mediated transport networks.

These transport corridors, essential for road and rail travel, appear to act as “invasion highways” for the unintentional passive dispersal (e.g. though soil or plants contaminated with eggs, larvae or adult stages) of invasive species, a phenomenon also observed in other studies^34,35^. Similar transport-corridor–driven expansions have been hypothesized for other invasive insects such as the brown marmorated stink bug *Halyomorpha halys*^36^ and the tiger mosquito *Aedes albopictus*^37^. Beyond *P. japonica*, comparative evidence indicates that multiple independent introductions via global trade and transport hubs may be common for invasive insects. For example, *H. halys* has shown repeated introductions across continents^38^. Similarly, *Drosophila suzukii* has probably repeatedly been reintroduced into Europe^39^.

The genetic evidence from this study, including phylogenetic, demographic, and kinship analyses, combined with the geographic proximity of these areas to Ticino and Northern Italy, supports the conclusion that infestations in Basel and Valais are part of a broader regional spread. The fact that even individual captures can be linked to key transport routes highlights the critical role of human activity in shaping the distribution of invasive species. In this context, the recent detections of *P. japonica* in Freiburg (Germany)^27^, as well as in Mulhouse and Saint-Hippolyte (France) in 2025^3^, suggest that the beetle is already expanding beyond Switzerland and may be spreading along major highway corridors. Far from being a minor observation, this evidence underscores the urgent need to consider transportation networks in strategies for managing and mitigating the spread of invasive species. Recently, awareness campaigns have been actively implemented in Switzerland by both the cantonal plant health services and the Federal Office for Agriculture^40^, as raising awareness among travelers and passengers is particularly important since they can unknowingly contribute to the spread of *P. japonica*.

Genetic analysis of the Basel population revealed a distinct clade of six samples nested within a larger clade which includes the samples from Basel, Italy, Ticino, Valais, Luzern, Schwyz, and Uri. While the other Basel samples clustered within this larger clade, the six samples in the sister clade displayed high relatedness, consistent with a sibling relationship or recent common ancestry (Fig. 4, Supplementary Figure S3). This might reflect their collection context, as all the six individuals were captured with pheromone traps within 500 m on the same day (Joanna Weibel, Agroscope, personal communication), suggesting a shared parental origin. Alternatively, this clustering may be the result of a second introduction event involving a small number of founders, which would warrant further investigation.

In conclusion, our findings reveal two potential mechanisms of spread. In southern regions, particularly Ticino, *P. japonica* populations represent a direct and contiguous expansion from Italy, as indicated by their geographic proximity and temporal alignment. By contrast, populations further north, such as those from Kloten and Basel, may have originated from passive, human-mediated transport across greater distances and discontinuous dispersal. This interpretation is consistent with the IBD analyses, which did not support a pattern of gradual range expansion in the invaded areas.

The persistent spread of *P. japonica* across the country illustrates the challenges of managing this invasive species, particularly given its high dispersal capacity. Furthermore, climate change is expected to intensify these challenges, as projections indicate that the suitable range for *P. japonica* in Switzerland will shift northward as rising temperatures create more favorable conditions for its establishment and survival^2^. Enhancing containment and control efforts requires a deeper understanding of the environmental and landscape factors that drive *P. japonica* movement and spread, which may enable targeted management in high-risk areas. By advancing our understanding of these invasion dynamics, this study provides valuable insights into the biology of invasive insect species and offers practical guidance for improving management strategies. Specifically, it highlights the need for enhanced biosecurity measures at airports to reduce the risk of new introductions or spread from infested areas through international air travel. It also emphasizes the importance of intensified regional surveillance, including monitoring spread of *P. japonica* along transportation routes and the importance of raising public awareness.

The findings support predictive models and provide important knowledge for the refinement of preventative strategies, and improvement of pest management practices. For example, raising awareness at high-risk spread sites, such as infested regions frequently visited by tourists from non-infested areas, and implementing targeted monitoring at key transport points can enhance early detection and control efforts. While addressing specific challenges of a location (in this case Switzerland), the results offer valuable insights for the global study of invasive species. By bridging academic research and practical applications, this study lays a foundation for future research and assists pest control strategies at both regional and global levels.

## Material and methods

### Collection of individuals and DNA extraction

In 2023 and 2024, 16 collections of *Popillia japonica* adults were established representing 14 sites across Switzerland, with two sites sampled more than once (Table 1, Fig. 1). These included 10 sites located in the Cantons of Ticino, Basel, Valais, and Zürich, corresponding to established outbreaks, and four sites in the Cantons of Luzern, Schwyz, and Uri, where isolated individuals were captured.

The global map and the map illustrating the collection sites for *P. japonica* beetles in Switzerland, also including samples from Ticino referenced in the study by Funari et al.^11^, were generated using the packages ggplot2 3.5.1^41^, sf 1.0–15 [https://r-spatial.github.io/sf/;^42^, rnaturalearth 1.0.1^43^ and raster 3.6–26^44^. Invasion areas were delineated based on publicly available surveillance updates from the Swiss Federal Office for Agriculture [FOAG;^45^] and the IPM-Popillia project^46^.

Following collection using pheromone-baited funnel traps combining floral attractants and synthetic lures, and direct sampling from host plants, the beetles were transported to the laboratory, frozen, and stored at −80 °C. Male individuals were dissected, and DNA was extracted from testes and aedagus tissue using the Wizard Genomic DNA Purification Kit (Promega, WI, USA). DNA quality was assessed visually on 1% agarose gels and quantified using a Qubit 2.0 fluorometer (HS dsDNA Kit, Thermo Fisher Scientific) and a NanoDrop Microvolume Spectrophotometer (Thermo Fisher Scientific, Waltham, MA, USA).

### Library preparation and sequencing

Following additional DNA quality and integrity check using a TapeStation gDNA Screen Tape (Agilent, Santa Clara, CA, USA), samples were sequenced at Macrogen (Amsterdam, The Netherlands). Consistently with Funari et al.^11^, library preparation was performed using the TruSeq DNA PCR-Free Kit (Illumina, San Diego, CA, USA) for high-quality DNA samples (Supplementary Table S1). For samples with lower DNA concentrations, the TruSeq Nano Kit (Illumina, San Diego, CA, USA) was employed, which includes an additional PCR amplification step (Supplementary Table S1). Whole genome resequencing of the libraries was performed using the NovaSeq 6000 platform with 150 bp paired-end reads, targeting 20 gigabases of sequence data per individual. A total of 119–274 million reads were obtained per sample with no noticeable differences among the two library types. The raw sequencing data (42 individuals from 14 collection sites, with 1 to 9 individuals sampled from each site) have been deposited in ENA under accession number PRJEB84386.

### Sequence quality control, variant calling, and SNP filtering

Data analysis follows Funari et al.^11^ with minor modifications. Raw reads were quality checked in FastQC 0.11.9^47^, trimmed in fastp 0.23.2^48^ and then remapped to the *P. japonica* reference genome [GCA_040143775.1;^49^ using bbmap 35^50^. Duplicate reads were removed with Picard 2.2.4 (http://broadinstitute.github.io/picard), and non-matching read pairs were filtered out using SAMtools 1.11^51^. Variant calling was performed using the multiallelic caller in BCFtools 1.13^51^ *ex novo* on the combined 83 individuals from Funari et al.^11^ and 42 individuals newly sequenced in this study. Calling was restricted to 14,989,716 genomic positions identified as variable in Funari et al. ^11^ that exclude sites on short contigs (< 1 kbp) or contigs with abnormal coverage (± 3 SD from the mean), sites within repetitive regions, sites ≤ 5 bp from indels, and sites with more than two alleles.

Following extensive data visualization to identify sensible thresholds, filtering was applied retaining SNPs with a minor allele frequency (MAF) > 0.01 (corresponding to at least three chromosomes), mean depth between 15× and 52x, site quality > 50 and missing data < 5% (parameters were adapted from Funari et al.^11^). Individual-level metrics—including mean depth, missing sites, and heterozygosity—were examined to identify potential outliers. Linkage disequilibrium pruning was then conducted with PLINK 1.90b6.21^52^ using a 50 kb window, 10 kb step size, and r2 threshold of 0.1, yielding a final dataset of unlinked SNPs, which was used for all analyses unless otherwise specified.

### Population genomic structure

Population genomic structure analyses were conducted following the protocols described by Funari et al.^11^, with minimal modifications. Analyses were performed on the SNP dataset, which included the 42 *P. japonica* samples collected in Switzerland for this study and the 83 individuals analyzed in Funari et al.^11^. The R package VcfR 1.15.0^53^ was used to import vcf files into R. To summarize genetic variation and visualize patterns of genetic differentiation among the 125 *P. japonica* individuals, a preliminary principal component analysis was constructed from the unlinked SNPs dataset with the R packages ADE4 1.7–18^54^, ADEGENET 2.1.5^55^ and ggplot2 3.5.1^41^. To examine individual admixture proportions and explore population genetic structure within and among *P. japonica* collections, a model-based clustering algorithm was implemented in ADMIXTURE v1.3.0^56^ assuming K values from 1 to 10. The most likely number of clusters was identified based on the lowest cross validation error; however, results are reported for K ranging from 1 to 10 as they may be informative at different levels. Hence, a range of K-values were analyzed and visually inspected^57,58^. Q-matrices were imported into R and assignment membership were constructed with the R package ggplot2 3.5.1^41^.

The possibility of isolation by distance (IBD) was evaluated at the population level across all main genetic groups identified by previous analyses, corresponding to those reported by Funari et al.^11^, with the addition of the Swiss samples (South Japan, North/Central Japan, USA and Canada, São Miguel and São Jorge – Azores, Italy/Ticino/Valais/Luzern/Uri/Schwitz, Basel – Switzerland, and Kloten – Switzerland). To specifically assess patterns within the invasive range, thereby avoiding a potential bias due to large geographic distances, the analysis was repeated excluding populations from Japan. Nei’s genetic distance^59^ was calculated using the *dist.genpop* function in the R package ADEGENET 2.1.5^55^, and Mantel tests^60^ were conducted, to evaluate the correlation with geographic distances, with the R package vegan^61^, employing Pearson’s correlation coefficient and 999 permutations.

Kinship analyses were conducted within and among the same genetic groups described above using PLINK 2.0^52^ to estimate mean φ pairwise coefficients via the KING-robust algorithm^62^. The heatmap was generated in R using ggplot2 v3.5.1^41^. Consistent with recommendations from Manichaikul et al.^62^, the full dataset of unpruned SNPs was utilized to maximize the precision of kinship estimates.

### Phylogenetic analyses

The vcf2phylip 2.9 program^63^ was used to convert the variant call format (VCF) into a phylip (.phy) file compatible with IQTREE. To infer evolutionary relationships of the 125 *P. japonica* individuals, including samples established in the frame of this study and samples from Funari et al.^11^, a maximum-likelihood (ML) phylogenetic analysis was conducted using IQ-TREE 2.3.6^64^. Support for the resulting phylogeny was assessed using 2,000 bootstrap replicates, ensuring robustness in the inference of branch confidence. The ModelFinder module within IQ-TREE was utilized to identify the most appropriate model of nucleotide substitution, optimizing for the best fit according to the Bayesian Information Criterion (BIC). A phylogenetic tree was visualized with the R packages ggplot2 3.5.1^41^ and ggtree 3.12.0^65^.

### Demographic inference

Demographic modeling to test the origin of Swiss populations, namely from Basel and Kloten, was conducted using Fastsimcoal 2^66,67^ on Site Frequency Spectra (SFS) derived from genomic data in VCF format. The SFS was generated with easySFS (https://github.com/isaacovercast/easySFS), optimizing SNP retention while minimizing missing data. We modelled 32 invasion scenarios across six main genetic groups, defined in the *Population genomic structure* section. Samples as single catches from Luzern, Uri, and Schwyz were excluded. Samples from Japan were excluded as not related to the European invasion based on prior analyses. The origins of USA + Canada, São Miguel, São Jorge, and Italy + Ticino + Valais were fixed based on historical records and the results of population structure analyses from Funari et al.^11^. For Basel and Kloten, we initially tested single-source invasion scenarios and then focused on admixture models involving USA, Italy + Ticino, and/or Basel as sources for Kloten, in line with the order of population origin, excluding the Azorean populations for efficiency based on preliminary tests of single-source invasion models. Invasion dates for USA + Canada, São Miguel, São Jorge, and Italy + Ticino + Valais were set 1–10 years flat prior to their first recorded reports^11^, assuming one generation per year^4^. For Basel and Kloten, where populations are closely monitored, invasion dates were modeled as two years prior to their first official reports, reflecting the likelihood of early detection. The four ‘ancestral’ populations were modeled in two stages. The first stage simulated a bottleneck event at the time of invasion, with bottleneck durations ranging from 1 to 6 generations and population sizes ranging from 10 to 500 individuals. The second stage modeled stable populations, which sizes ranged from 10 to 10,000,000 individuals. The two ‘contemporary’ populations (Basel and Kloten), given their recent introduction, were modeled in one step with population sizes ranging from 10 to 10,000 individuals. Gene flow was assumed absent in all scenarios. Each scenario was tested using 50 independent runs, 40 conditional maximization algorithm cycles, and 500,000 simulations per run. Model selection was based on the Akaike Information Criterion (AIC) applied to the top-performing runs of all 32 scenarios, accounting for model complexity.

## Supplementary Information

Supplementary Information.

## Data Availability

Sequences were deposited in the European Nucleotide Archive (ENA) under BioProject PRJEB accessions PRJEB84386.
