# Supplementary material for: Population genomics identifies Italian and North American origins of Popillia japonica in Switzerland
Source: Sci Rep. 2026 Feb 12;16:8445. doi: 10.1038/s41598-026-39440-4 (PMC12972101; doi:10.1038/s41598-026-39440-4)
Supplement: Supplementary file 1 — Supplementary Information. [file 41598_2026_39440_MOESM1_ESM.docx]

Population genomics identifies Italian and North American source populations of *Popillia japonica* in Switzerland

Chiara Pedrazzini^1*^, Rebecca Funari^2^, Claudio Cucini^2, 3^, Francesco Nardi^2, 3^, Giselher Grabenweger^4^, Franco Widmer^1^, Jürg Enkerli^1^

^1^Molecular Ecology, Agroscope, Zürich, Switzerland

^2^Dipartimento di Scienze della vita, Università di Siena, Siena, Italy

^3^NBFC, National Biodiversity Future Center, Palermo, Italy

^4^Extension Arable Crops, Agroscope, Zürich, Switzerland

Keywords: Japanese beetle, population genomics, genetic structure, biological invasion

*Corresponding author

Table of Contents

| **Supplementary Material** | **Page** |
| --- | --- |
| Supplementary Table S1 | 2 |
| Supplementary Table S2 | 3-4 |
| Supplementary Figure S1 | 5 |
| Supplementary Figure S2 | 6 |
| Supplementary Figure S3 | 7 |
| Supplementary Figure S4 | 8 |
| Supplementary Figure S5 | 9 |

**Supplementary Table S1.** Label, canton of origin, and sequencing technology used for library construction in this study.

| **New ID** | **Canton** | **Year of collection** | **Library preparation** |
| --- | --- | --- | --- |
| ZH_1 | Zürich | 2023 | TruSeq DNA PCR-Free |
| ZH_5 | Zürich | 2023 | TruSeq DNA PCR-Free |
| ZH_7 | Zürich | 2023 | TruSeq DNA PCR-Free |
| ZH_8 | Zürich | 2023 | PCR-based kit (TruSeq Nano) |
| ZH_9 | Zürich | 2023 | PCR-based kit (TruSeq Nano) |
| ZH_11 | Zürich | 2023 | PCR-based kit (TruSeq Nano) |
| ZH_12 | Zürich | 2023 | PCR-based kit (TruSeq Nano) |
| ZH_13 | Zürich | 2023 | PCR-based kit (TruSeq Nano) |
| ZH_25 | Zürich | 2024 | TruSeq DNA PCR-Free |
| ZH_26 | Zürich | 2024 | TruSeq DNA PCR-Free |
| ZH_27 | Zürich | 2024 | TruSeq DNA PCR-Free |
| ZH_28 | Zürich | 2024 | TruSeq DNA PCR-Free |
| ZH_29 | Zürich | 2024 | TruSeq DNA PCR-Free |
| ZH_30 | Zürich | 2024 | TruSeq DNA PCR-Free |
| ZH_31 | Zürich | 2024 | TruSeq DNA PCR-Free |
| ZH_32 | Zürich | 2024 | TruSeq DNA PCR-Free |
| BL_2 | Basel-Landschaft | 2023 | PCR-based kit (TruSeq Nano) |
| BL_4 | Basel-Landschaft | 2024 | TruSeq DNA PCR-Free |
| BL_5 | Basel-Landschaft | 2024 | TruSeq DNA PCR-Free |
| BL_6 | Basel-Landschaft | 2024 | TruSeq DNA PCR-Free |
| BL_10 | Basel-Landschaft | 2024 | PCR-based kit (TruSeq Nano) |
| BL_12 | Basel-Landschaft | 2024 | PCR-based kit (TruSeq Nano) |
| BL_13 | Basel-Landschaft | 2024 | PCR-based kit (TruSeq Nano) |
| BL_14 | Basel-Landschaft | 2024 | TruSeq DNA PCR-Free |
| BL_16 | Basel-Landschaft | 2024 | TruSeq DNA PCR-Free |
| BL_18 | Basel-Landschaft | 2024 | TruSeq DNA PCR-Free |
| VS_1 | Valais | 2024 | PCR-based kit (TruSeq Nano) |
| VS_2 | Valais | 2024 | PCR-based kit (TruSeq Nano) |
| VS_4 | Valais | 2024 | PCR-based kit (TruSeq Nano) |
| VS_6 | Valais | 2024 | TruSeq DNA PCR-Free |
| VS_9 | Valais | 2024 | TruSeq DNA PCR-Free |
| VS_10 | Valais | 2024 | PCR-based kit (TruSeq Nano) |
| VS_11 | Valais | 2024 | PCR-based kit (TruSeq Nano) |
| VS_12 | Valais | 2024 | PCR-based kit (TruSeq Nano) |
| TI_7 | Ticino | 2023 | TruSeq DNA PCR-Free |
| TI_10 | Ticino | 2023 | TruSeq DNA PCR-Free |
| TI_11 | Ticino | 2023 | TruSeq DNA PCR-Free |
| TI_13 | Ticino | 2023 | PCR-based kit (TruSeq Nano) |
| UR_1 | Uri | 2024 | PCR-based kit (TruSeq Nano) |
| LU_2 | Luzern | 2024 | PCR-based kit (TruSeq Nano) |
| LU_1 | Luzern | 2024 | TruSeq DNA PCR-Free |
| SZ_1 | Schwyz | 2024 | PCR-based kit (TruSeq Nano) |

**Supplementary Table S2.** Statistical model estimates for the demographic inference analyses. The best-supported model, according to Akaike Information Criterion, is shown in bold. Abbreviations: MaxEstLhood, Maximum Estimated Likelihood; MaxObsLhood, Maximum Observed Likelihood; k, number of estimated parameters; AIC, Akaike Information Criterion; relative_Lhood, Relative Likelihood.

| **Model** | **MaxEstLhood** | **MaxObsLhood** | **Best run** | **k** | **Best AIC** | **BEST_ML** | **deltaAIC** | **relative_Lhoods** | **Akaike_weights** |
| --- | --- | --- | --- | --- | --- | --- | --- | --- | --- |
| **model1** | **-972.708** | **-956.703** | **run7** | **16** | **4511.486** | **-972.708** | **0.000** | **1.000** | **0.728** |
| model30 | -972.793 | -956.703 | run33 | 18 | 4515.877 | -972.793 | 4.391 | 0.111 | 0.081 |
| model24 | -973.292 | -956.703 | run3 | 17 | 4516.175 | -973.292 | 4.689 | 0.096 | 0.070 |
| model26 | -973.311 | -956.703 | run40 | 17 | 4516.263 | -973.311 | 4.777 | 0.092 | 0.067 |
| model31 | -972.97 | -956.703 | run24 | 18 | 4516.692 | -972.97 | 5.207 | 0.074 | 0.054 |
| model16 | -978.285 | -956.703 | run1 | 16 | 4537.169 | -978.285 | 25.683 | 2.65E-06 | 1.93E-06 |
| model23 | -978.362 | -956.703 | run12 | 17 | 4539.524 | -978.362 | 28.038 | 8.16E-07 | 5.94E-07 |
| model32 | -978.355 | -956.703 | run39 | 18 | 4541.491 | -978.355 | 30.005 | 3.05E-07 | 2.22E-07 |
| model15 | -979.941 | -956.703 | run42 | 16 | 4544.795 | -979.941 | 33.309 | 5.85E-08 | 4.26E-08 |
| model13 | -980.29 | -956.703 | run17 | 16 | 4546.402 | -980.29 | 34.916 | 2.62E-08 | 1.91E-08 |
| model3 | -980.356 | -956.703 | run6 | 16 | 4546.706 | -980.356 | 35.220 | 2.25E-08 | 1.64E-08 |
| model25 | -980.339 | -956.703 | run27 | 17 | 4548.628 | -980.339 | 37.142 | 8.60E-09 | 6.27E-09 |
| model22 | -980.349 | -956.703 | run26 | 17 | 4548.674 | -980.349 | 37.188 | 8.41E-09 | 6.12E-09 |
| model14 | -982.587 | -956.703 | run43 | 16 | 4556.980 | -982.587 | 45.494 | 1.32E-10 | 9.62E-11 |
| model4 | -982.611 | -956.703 | run28 | 16 | 4557.091 | -982.611 | 45.605 | 1.25E-10 | 9.11E-11 |
| model21 | -982.647 | -956.703 | run26 | 17 | 4559.257 | -982.647 | 47.771 | 4.23E-11 | 3.08E-11 |
| model2 | -983.768 | -956.703 | run32 | 16 | 4562.419 | -983.768 | 50.933 | 8.71E-12 | 6.34E-12 |
| model6 | -992.387 | -956.703 | run17 | 16 | 4602.111 | -992.387 | 90.625 | 2.09E-20 | 1.53E-20 |
| model12 | -998.356 | -956.703 | run4 | 16 | 4629.599 | -998.356 | 118.113 | 2.25E-26 | 1.64E-26 |
| model11 | -1000.705 | -956.703 | run3 | 16 | 4640.417 | -1000.705 | 128.931 | 1.01E-28 | 7.33E-29 |
| model19 | -1003.111 | -956.703 | run30 | 16 | 4651.497 | -1003.111 | 140.011 | 3.95E-31 | 2.88E-31 |
| model20 | -1007.123 | -956.703 | run1 | 16 | 4669.973 | -1007.123 | 158.487 | 3.85E-35 | 2.80E-35 |
| model5 | -1009.051 | -956.703 | run28 | 16 | 4678.852 | -1009.051 | 167.366 | 4.54E-37 | 3.31E-37 |
| model8 | -1022.824 | -956.703 | run25 | 16 | 4742.279 | -1022.824 | 230.793 | 7.66E-51 | 5.58E-51 |
| model10 | -1023.288 | -956.703 | run5 | 16 | 4744.415 | -1023.288 | 232.930 | 2.63E-51 | 1.92E-51 |
| model7 | -1025.186 | -956.703 | run37 | 16 | 4753.156 | -1025.186 | 241.670 | 3.33E-53 | 2.42E-53 |
| model9 | -1033.957 | -956.703 | run28 | 16 | 4793.548 | -1033.957 | 282.062 | 5.64E-62 | 4.11E-62 |
| model18 | -1035.885 | -956.703 | run14 | 16 | 4802.427 | -1035.885 | 290.941 | 6.65E-64 | 4.85E-64 |
| model17 | -1039.349 | -956.703 | run14 | 16 | 4818.379 | -1039.349 | 306.893 | 2.29E-67 | 1.66E-67 |
| model27 | -1039.478 | -956.703 | run6 | 17 | 4820.973 | -1039.478 | 309.487 | 6.25E-68 | 4.55E-68 |
| model29 | -1043.399 | -956.703 | run46 | 17 | 4839.030 | -1043.399 | 327.544 | 7.49E-72 | 5.46E-72 |
| model28 | -1056.244 | -956.703 | run43 | 17 | 4898.183 | -1056.244 | 386.697 | 1.07E-84 | 7.80E-85 |


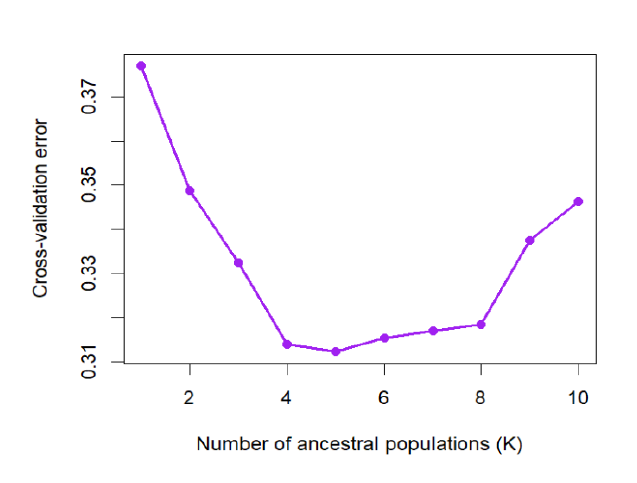
​​

​​**Supplementary Figure S1.** Cross-entropy results of ADMIXTURE runs for *Popillia japonica*. The run with the lowest cross-entropy value (K=5) represents the optimal population clustering scenario.

**
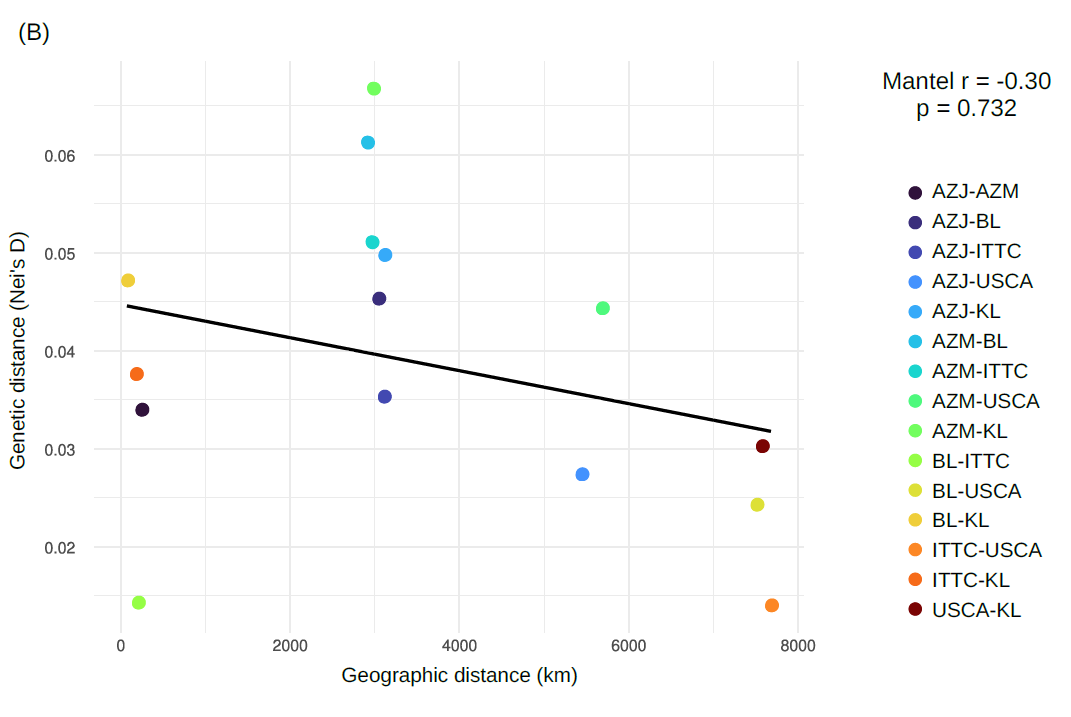

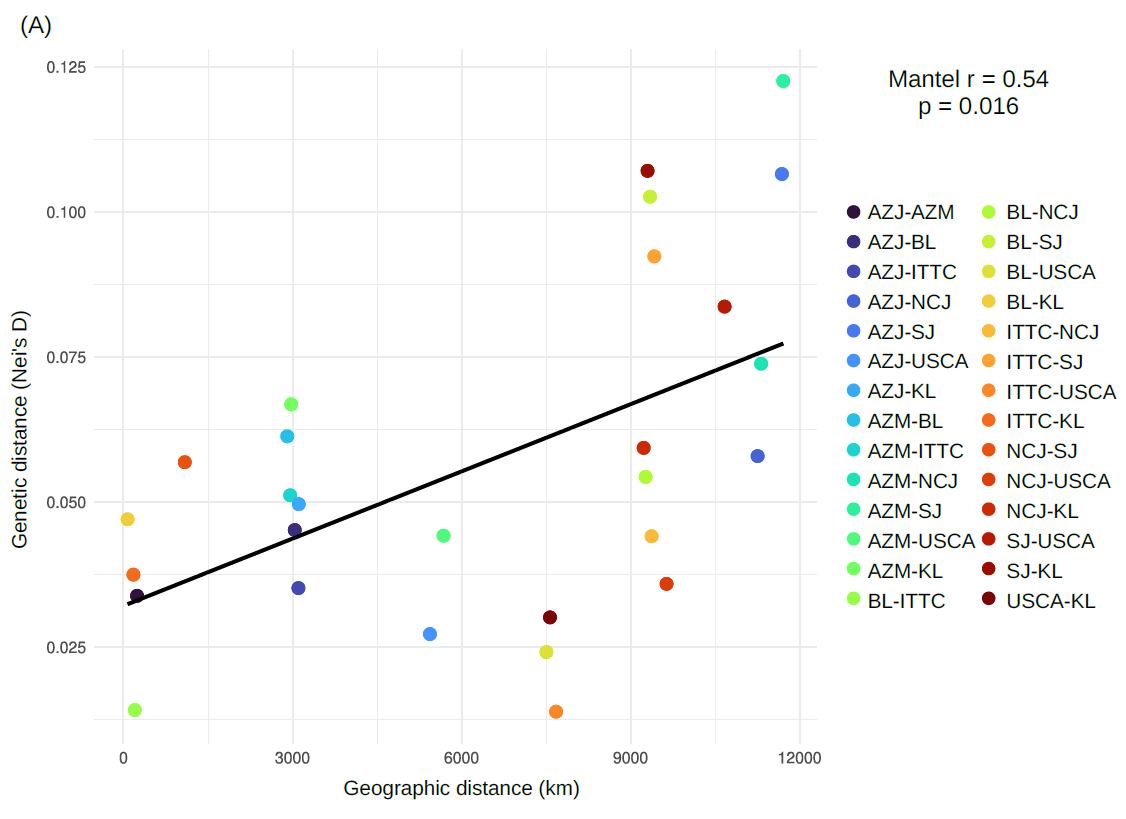
**

​​**Supplementary Figure S2.** Correlation between genetic and geographic distances based on Mantel tests. (A) Isolation-by-distance analysis including all genetic groups. (B) Isolation-by-distance analysis excluding populations from Japan. p, p-value for significance.


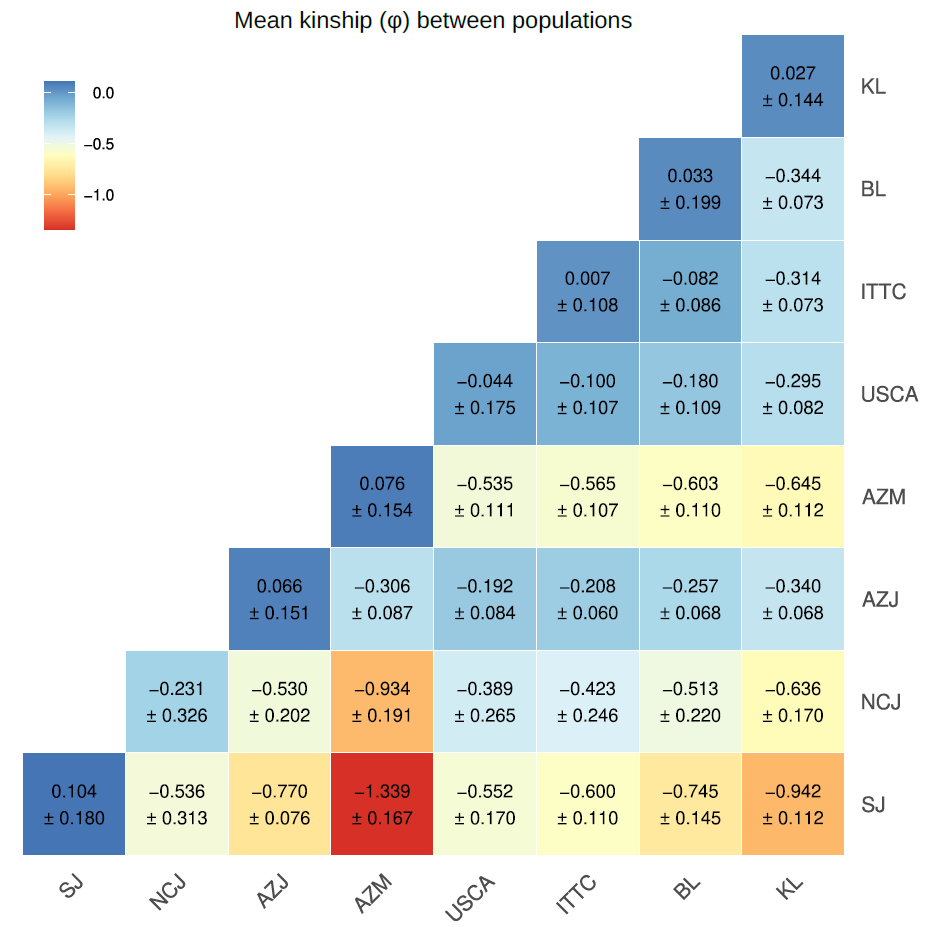


​​​​

**Supplementary Figure S3.** Heatmap of the kinship matrix obtained using the KING-robust algorithm implemented in PLINK 2.0, based on the unpruned SNP dataset. The heatmap was generated in R using ggplot2 v3.5.1 (https://ggplot2.tidyverse.org/). Abbreviations: SJ, South Japan; NCJ, North/Central Japan; USCA, USA and Canada; AZJ, São Jorge (Azores); AZM, São Miguel (Azores); ITTC, Italy, Ticino, Valais, Luzern, Uri, and Schwyz; BL, Basel; KL, Kloten.


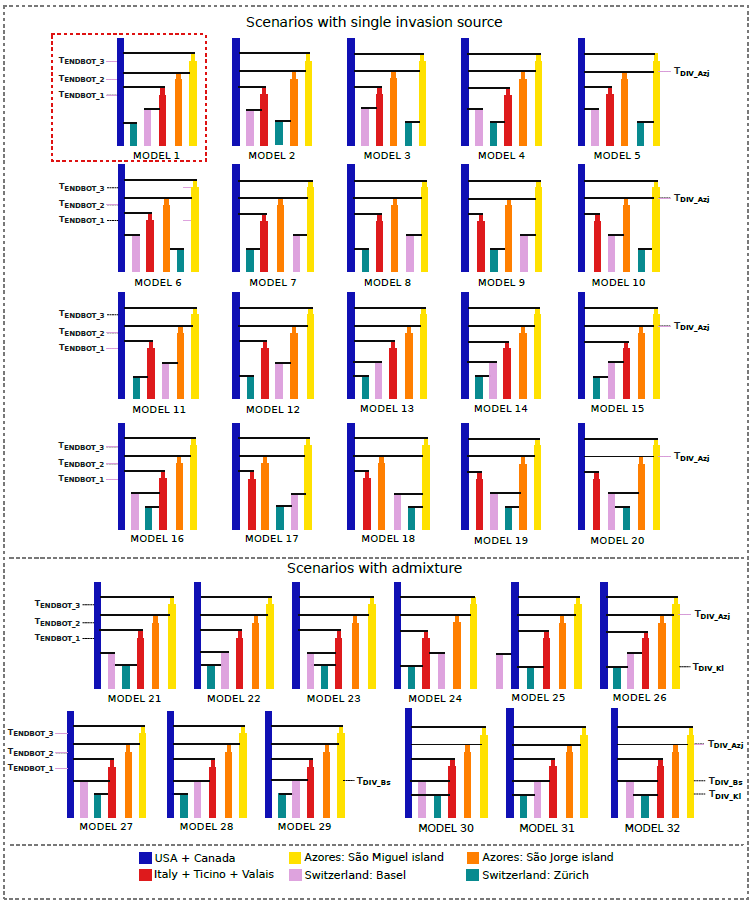


**Supplementary Figure S4.** All the potential invasion scenario models investigated by demographic inference analyses. Fastsimcoal2 models tested to study the invasion pathways of *Popillia japonica* in Basel and Kloten (Switzerland). Model 1, in the red box, was the best model according to Akaike Information Criterion. T_DIV_, indicates the divergence time of the admixed lineages. _Azj_, São Jorge (Azores); _Bs_, Basel (Switzerland); _Kl_, Kloten (Switzerland). T_ENDBOT_: Bottleneck End Time.


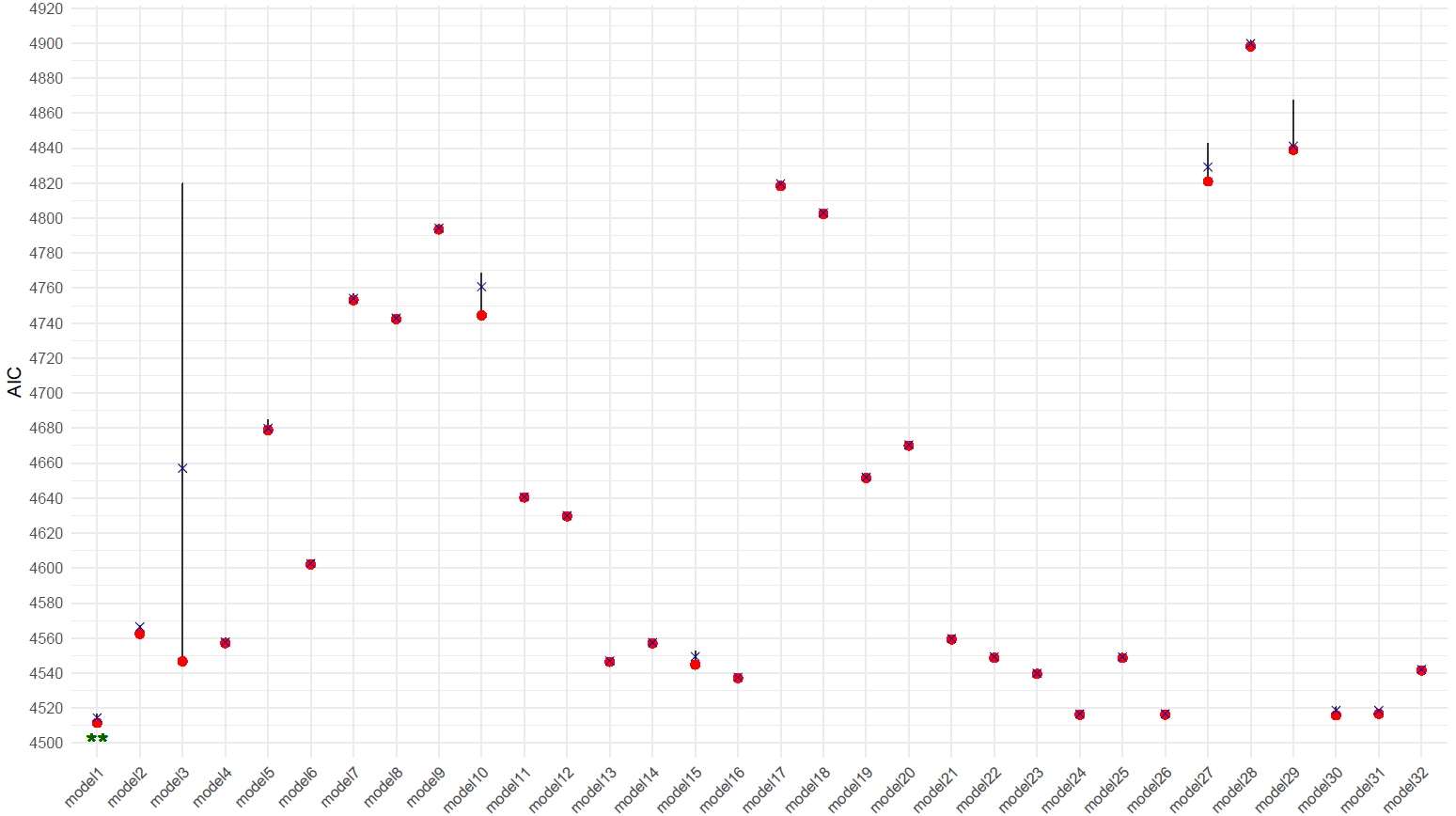


**Supplementary Figure S5.** Distribution of Akaike Information Criterion (AIC) values for each model and subset tested in the step-by-step demographic analysis using FastSimcoal2. Red dots indicate the run with the lowest (i.e., best) AIC value. A double asterisk (**) denotes the best-fitting model based on AIC. A blue x indicates the mean.
